# General Meeting of the Academy of Sciences, Belles Lettres, and Arts, of Dijon; Held the 19th Fructidor, Year 10

**Published:** 1803-04-01

**Authors:** 


### General Meeting *of the* Academy *of* Sciences, Belles Lettres, *and* Arts, *of* Dijon; *held the* 19*th Fructidor, Year* 10.

At this Meeting the above title was adopted as a more proper denomination, and better suited to recall to our memory the illustrious society to which the present has succeeded.

In the report made of the labours of its members, we remark an account of the phenomenon of Scintillation produced by the concussion of carbonified wood. Three explosions had taken place in the powder mills of Vonges in the space of four months, notwithstanding every precaution being used to prevent it. In consequence of this remarkable repetition, Cit. Lemaitre, inspector general, was ordered to repair to the spot, and enquire into the cause of this accident. The inspector general, already known to the world as the author of many interesting memoirs in Natural History, &c. made a number of experiments, in order to fulfill the object of his mission. The reporter, Cit. Lischever, was present at many of them, and to his account the Academy is indebted for a knowledge of the singular phenomenon of striking fire, on the shock of carbonified wood with any other wood. For a more detailed account, it is necessary to read the history of the experiments, which prove this fact in the most incontestable manner; it realises the suspicions already conceived of the danger of using charcoal in sticks, in the fabrication of gunpowder. Cit. Lischever terminates his Memoir with the following reflection:

"Light and heat, when disengaged from combustible bodies, being so much the more abundant as the combination of oxygen with the body is greater in a given space of time, it should seem from the circumstances of the phenomenon just related, that a small degree of heat only is necessary to produce the combination of oxygen with charcoal, and the combustion of the latter."

The

The Academy has proposed the following question as the subject of a prize for the ensuing year:

"Catarrhal fevers are become more frequent than they have ever been. Inflammatory fevers are become more rare. Bilious fevers are less frequent. It is proposed to ascertain the causes which have given rise to this revolution in climate and temperament."

The value of the prize is 500 livres, and the contest is open to every one but members of the Academy.

Bilious and inflammatory affections, which stamped a character on most of the acute diseases of which the ancients have transmitted a faithful account, have evidently given way to the pituitous or catarrhal fever. Diseases of this order are in fact much less common in our days than formerly. It was about the middle of the fifteenth century that they took on that train of symptoms which at present characterize them; and they have been since observed, at different periods, to run over many countries of Europe and give rise to many epidemical diseases more or less mortal; such were those of the years 1775 and 1780. Such a change occurring in the system of diseases which afflict mankind, depend no doubt on the co-operation of a variety of causes as well physical as moral. It would be desirous to determine the description of individuals particularly subject to these diseases, and whether or not they are those of weak constitutions either natural or acquired. Do we not daily observe that women, children, and the aged, are more particularly attacked? Struck by these considerations, and desirous to contribute all in their power to throw light on a subject of so much general importance, the ancient Academy proposed this as the subject for the prize at their public Meeting, August 25, 1788. The Memoirs which were delivered in consequence, were not judged to answer completely the intentions of the Academy; the revolution suspended the further prosecution of the subject, and the present Academy, actuated by the same motives as the former, have renewed the question as a subject of general medical interest.

Memoirs written in the French or Latin languages to be addressed post paid to Cit. Vallot, M. D. Secretary of the Academy, before the 1st Messidor, An. 12.

